# Effect of sympathetic autonomic stress from the cold pressor test on left ventricular function in young healthy adults

**DOI:** 10.14814/phy2.13985

**Published:** 2019-01-18

**Authors:** Simiat O. Elias, Reina E. Ajayi

**Affiliations:** ^1^ Department of Physiology Lagos State University College of Medicine Ikeja, Lagos Nigeria

**Keywords:** Autonomic stress, cold pressor test, left ventricular function

## Abstract

There is a dearth of studies investigating the effect of sympathetic activation on left ventricular function. This study aimed to investigate the effect of sympathetic autonomic stress on left ventricular function in young healthy adults. Fifty‐six normotensive healthy participants (age 23.55 ± 3.82 years) took part in the study after giving informed consent. After obtaining baseline measurements, heart rate (HR), blood pressure (BP), peripheral saturation of oxygen (SpO2) and left ventricular function (assessed by means of ejection fraction (EF) obtained by transthoracic 2‐D echocardiography) were determined before and following sympathetic activation using cold pressor test (CPT). Exposure to CPT led to significant increase (*P* < 0.0001) in HR (70.4 ± 10.7 bpm to 91.6 ± 14.8 bpm), SBP (118 ± 8 mmHg to 138 ± 14 mmHg) and DBP (71 ± 7 mmHg to 91 ± 11 mmHg). Participants’ EDV (101.1 ± 15.8 ml to 104.2 ± 19.3 mL), ESV (38.7 ± 9.1 mL to 40.3 ± 11.6 mL), SpO2 (99.5 ± 0.79% to 99.5 ± 0.77%) and EF (61.9 ± 5.9% to 60.9 ± 6.4%) were only slightly changed (*P* > 0.05). However, cardiac output (4.3 ± 0.9 L/min to 5.4 ± 1.4 L/min) and cardiac index (3.7 ± 0.8 L/min per m^2^ to 4.5 ± 1.4 L/min per m^2^) increased significantly (*P* < 0.0001). We conclude that sympathetic stress induced by cold pressor test has marginal effect on ejection fraction and fractional shortening while increasing cardiac output and cardiac index in young healthy adults.

## Introduction

The cardiovascular system is prone to being affected by stress caused by many factors experienced in modern life. Stress elicits changes in sympathetic–parasympathetic balance (Brotman et al. 2007) which might negatively affect the cardiovascular system acutely by precipitating myocardial infarction (Lucinda et al. 2015), left ventricular dysfunctions (Barclay and Vega 2005; Ramaraj 2007) and arrhythmias (Ziegelstein 2007). Stress activates the sympathetic function of the autonomic nervous system causing an increase in blood pressure (Trapp et al. 2014), heart rate (Ayada et al. 2015) and cardiac output (Von Baeyer et al. 2005), and release of stress hormones. Indeed, this can lead to a series of heart diseases which include arrhythmias and sudden cardiac death. Left ventricular function is usually established by computing ejection fraction (Foley et al. 2012) which in healthy people ranges between 50% and 75%. Interest in assessing cardiac function has resulted from advances in understanding the physiology of cardiac muscle contraction using repetitive and noninvasive techniques. Ultrasonic transthoracic echocardiography has been used to provide quantitative information about left ventricular function by derivation of ventricular volumes and ejection fraction.

The cold pressor test (CPT) is an experimental cold stimulus used clinically as a stress test to evaluate cardiac autonomic function and to assess left ventricular function (Wirch et al. 2006). Its vascular sympathetic response typically is an increased peripheral resistance and a sustained blood pressure increase (Cui et al. 2002). The effect on heart rate is variable being either an increase or remaining unchanged though the latter is thought to be a biphasic response with an initial rise in those subjects followed by a slow decline that may even return to the pretest level (Mourot et al. 2009). Ifuku et al. (2007) reported a decrease in heart rate and cardiac contractility of untrained subjects while total peripheral resistance and blood pressure increased. Acute stress response in young, healthy individuals may be adaptive and typically does not impose a health burden (Schneiderman et al. 2005). However, continuous stress can lead to a health condition especially in older or unhealthy individuals. Yalçin et al. (2011) reported that exposure to acute and chronic stress caused regional left ventricular hyper‐contractility and dysfunction especially in susceptible individuals. Similarly, Zhao et al. (2007), in animal model experiments, reported that chronic stress resulted in cardiac dysfunction and structural injury to the heart. The interaction between the left ventricle and the systemic arterial vasculature (ventriculararterial coupling) is an important determinant of left ventricular function (Chantler et al. 2008). We therefore hypothesized that autonomic stress through the CPT will have significant effect on left ventricular function in young adults and designed this study to test this. Relatively few studies have investigated the effects of the CPT on left ventricular function.

## Methods

### Ethical approval

The protocols for this study were approved (LREC/10/06/590) by the Health Research and Ethics Committee of the Lagos State University Teaching Hospital (LASUTH), Ikeja. The study was carried out in accordance with the latest revision of the *Declaration of Helsinki* except for registration in a database. All experiments were well‐tolerated by the participants.

### Participants

Fifty‐six (56) normotensive young healthy participants took part in the study after informed consent was obtained from them. The participants were informed of the protocol and details of the study before signing the consent form. Questionnaires were given to all of them where they stated if they had any family history of hypertension.

#### Inclusion Criteria

Healthy young adults aged 18 years to 35 years, and normotensive with a resting blood pressure <140/90 mmHg were continually enrolled into the study from Lagos State University College of Medicine (LASUCOM) and LASUTH community. They were nonsmokers with no history of smoking. They were nonobese with a body mass index (BMI) less than 30 kg/m^2^. They had no history of chest pain and they were not on any cardiovascular or respiratory medications.

#### Exclusion criteria

Individuals were excluded if their resting blood pressure was <110/60 mmHg; baseline plasma norepinephrine was high above the normal range or their enddiastolic volume and endsystolic volume below the normal range. Females in their menstrual flow, pregnant or on oral contraceptives were also excluded. Trained athletes were also excluded from this study, because they tend to have increased muscle mass (Maron and Pelliccia 2006).

### Study design

The study was carried out in the Research Laboratory of the Department of Physiology, Lagos State University College of Medicine (LASUCOM), Ikeja and the Echocardiography Laboratory of the Lagos State University Teaching Hospital (LASUTH), Ikeja. Participants were asked to refrain from caffeinecontaining beverages for at least three h, and alcohol beverages and exercise for at least four h prior to experiment (Hartwich et al. 2010). Upon arrival, participants were allowed to rest for 10 min before the commencement of the study. The procedure which had earlier been explained to them was reinforced prior to commencement of the tests.

### Experimental Procedure

Baseline measurements including the participant's height (m), measured to the nearest 0.1 m using a standard stadiometer (Seca 217) and body weight (kg) measured using a body weighing machine (Camry C200) were carried out. The participant was then asked to lie on the couch in a supine position, with their shoes taken off. The participant was connected to a Comen C80 patient monitor system (Shenzhen Comen Medical Instrument Co. Ltd, China) for the measurement baseline blood pressure (BP) using an automatic cycling noninvasive BP monitor and the standard oscillometric method. Heart rate (beat/min) of the participants was measured from Lead II of the 12‐lead ECG cable connected to the Patient Monitor after recording for 1 min at a speed of 25 mm/sec.

### Twodimensional echocardiographic assessment

Left ventricular function was assessed using a Vivid q Cardiovascular ultrasound machine (General Electric Medical System, Horten, Norway). All ultrasound assessments were made by REA, a trained sonographer with LASUTH, Ikeja. With participants lying in the left lateral decubitus position, a phasedarray transducer with a frequency of 3.5 MHz was connected to the 2‐Dimensional transthoracic echocardiography machine and placed over the left border of the sternum. The image of the heart was reflected on a gray scale picture on the monitor of the echocardiography machine. Left ventricular function (LVF) was measured by using the motionmode (M‐mode) technique obtained at the left parasternal long axis view (the first echocardiography view). An Mmode cursor was placed through the septal and posterior left ventricular walls just beyond the tip of the mitral leaflets. The internal left ventricular dimensions were measured between the endocardial border of the septum and the endocardial border of the posterior wall in systole and diastole (Lang et al. 2015). Left ventricular internal diameter in diastole and left ventricular end diastolic volume (LVEDV) were used as indices of preload (Wilson et al. 2010). Ejection fraction was used as an index of systolic function (Wilson et al. 2010).

### Measurement of peripheral capillary saturation oxygen

To determine whether the participants were performing Valsalva maneuver on exposure to the CPT, continuous measurement of peripheral capillary saturation oxygen (SpO2) of participants was carried out before and during exposure to the test using a Comen C80 adult finger clip SpO2 sensor, part of the Patient Monitor.

### Exposure of participants to the cold pressor test

Prior to the onset of this procedure, the participants were instructed to avoid performance of Valsalva maneuver or hyperventilating during the test. They were then asked to immerse their right foot up to the ankle for 1 min in ice slurry maintained at 4°C (Elias et al. 2014). With the foot still in the ice slurry, simultaneous measurement of BP, HR, SpO2 and assessment of left ventricular function were repeated as outlined above.

### Determination of plasma norepinephrine

Venous blood (3 mL) was drawn through a 5 mL syringe and placed into a chilled lithium heparinized bottle before and after the exposure of participants to the CPT. Plasma samples obtained were immediately stored in a −80°C freezer until analysis later. Norepinephrine was analyzed with a 2‐CAT ELISA ‐ Fast Track (Rocky Mountain Diagnostics Inc., Colorado Springs, Colorado) with a Norepinephrine kit (Sunlong Bio‐tech Co. Limited, China) following the manufacturer's instructions.

### Left ventricular hemodynamics

Mean Arterial Pressure, MAP (mmHg) was calculated as diastolic blood pressure (DBP) plus 1/3 pulse pressure. Stroke volume (mL) was calculated as LV end diastolic volume (mL) minus LV end systolic volume (mL). Cardiac output, $\dot{Q}$ (L/min) was calculated as the product of stroke volume, SV (mL) and heart rate, HR (beat/min). Total Peripheral Resistance (mmHg/L per min) was calculated as MAP/$\dot{Q}$. Cardiac Index (L/min per m^2^) was calculated as $\dot{Q}$/BSA.

### Statistical analyses

All statistical analyses were carried out using GraphPad Statistical software, Prism 5 for Windows (GraphPad Software, San Diego, California, USA). Data are expressed as mean ± Standard Deviation, (SD). Test for normality of distribution was carried out using the Shapiro‐Wilk test and where the test failed, the MannWhitney U test was carried out to detect differences between the nonparametric data. Differences in descriptive variables and cardiovascular responses before and after the CPT were determined using Student's paired *t*‐test. Correlation coefficients (r values) between the different studied parameters were sought using Spearman's rank correlation. Statistical significance was accepted at 95% confidence interval.

## Results

The characteristics of the participants and baseline cardiovascular measurements are shown in Table 1. Other baseline measurements of left ventricular function are shown in Table 2. Of the 56 subjects, 31(55.4%) had a positive history of hypertension while the remaining 25(44.6%) did not.

**Table 1:** Baseline characteristics and cardiovascular parameters

	X ± SD (range)
Characteristics of Participants	
Age (year)	23.55 ± 3.82 (18–34)
Weight (kg)	62.23 ± 8.36 (45–80)
Height (m)	1.68 ± 0.08 (1.52–1.86)
BMI (kg m^−2^)	21.9 ± 2.5 (16.3 ‐26.3)
BSA (m^2^)	1.7 ± 0.14 m^2^
Resting Hemodynamics	
SBP (mmHg)	118 ± 8 (100–130)
DBP (mmHg)	71 ± 7 (58–84)
MAP (mmHg)	88 ± 7 (75–100)
Heart rate (beats/min)	70.4 ± 10.7 (50–99)
SpO2 (%)	99.46 ± 0.79 (96–100)

*n* = 56; BMI, body mass index; BSA, body surface area; SBP, systolic blood pressure; DBP, diastolic blood pressure; MAP, mean arterial pressure; MAP, mean arterial pressure; SpO2, Peripheral capillary oxygen saturation; NE, Norepinephrine.

**Table 2:** Baseline left ventricular function parameters

	Mean ± SD (range)
Enddiastolic volume (mL)	101.12 ± 15.8 (63–133)
Endsystolic volume (mL)	36.67 ± 9.07 (22–59)
Fractional shortening (%)	33.28 ± 4.5 (26–46)
Ejection fraction (%)	61.90 ± 5.89 (51–75)
Stroke volume (mL)	61.58 ± 11.62 (36–102)
Cardiac output, $\dot{Q}$ (L/min)	4.29 ± 0.93 (2.4–6.5)
Cardiac index (L/min per m^2^)	3.68 ± 0.83 (2.3–5.3)
TPR (mmHg/L per min)	21.35 ± 5.06 (14–31)

TPR, Total Peripheral Resistance.

### Blood pressure response to the cold pressor test

**Figure 1:**
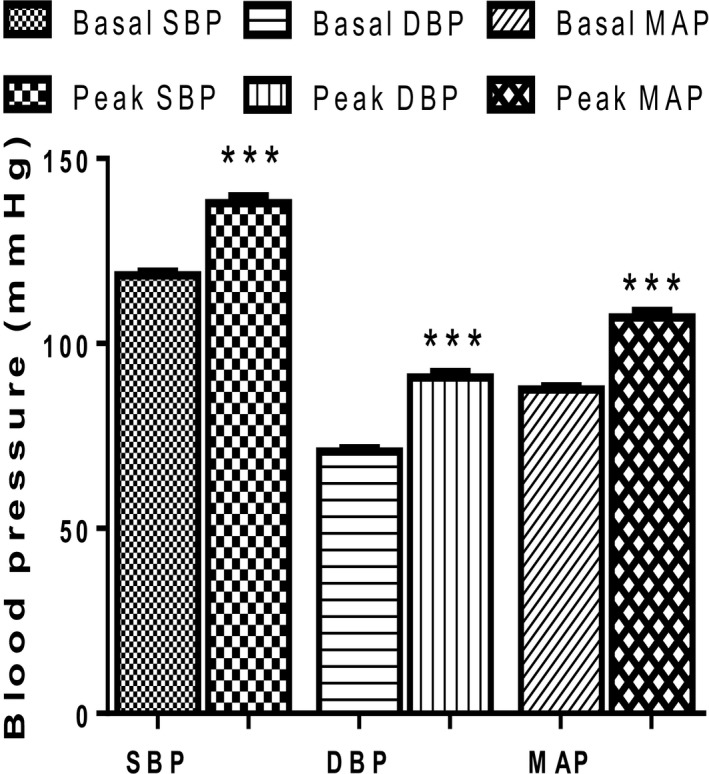
Blood pressure (BP) responses of participants before and after the cold pressor test (*n* = 56). *** = *P* < 0.0001 Peak BP versus Basal BP. SBP, systolic blood pressure; DBP, diastolic blood pressure; MAP, mean arterial pressure; CPT, cold pressor test; Basal, before exposure to CPT; Peak, after exposure to CPT.

Following exposure to the CPT, all the blood pressure parameters increased significantly (*P* < 0.0001): SBP from 118 ± 8 mmHg to 138 ± 14 mmHg; DBP from 71 ± 7 mmHg to 91 ± 11 mmHg and MAP from 88 ± 7 mmHg to 105 ± 18 mmHg (Fig. 1).

### Heart rate (HR) response to the cold pressor test

**Figure 2:**
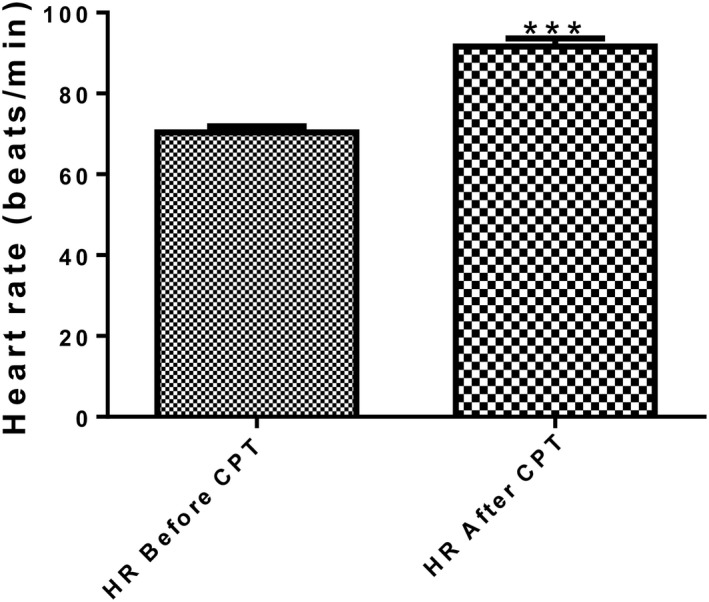
Heart rate of the participants before and after the cold pressor test (*n* = 56). *** = *P* < 0.0001 HR after CPT versus HR before CPT. HR, heart rate; CPT, cold pressor test.

When participants were exposed to the cold pressor test, heart rate increased significantly (*P* < 0.0001) as shown in Figure 2.

### Peripheral capillary oxygen saturation (SpO2) response to the cold pressor test

**Figure 3:**
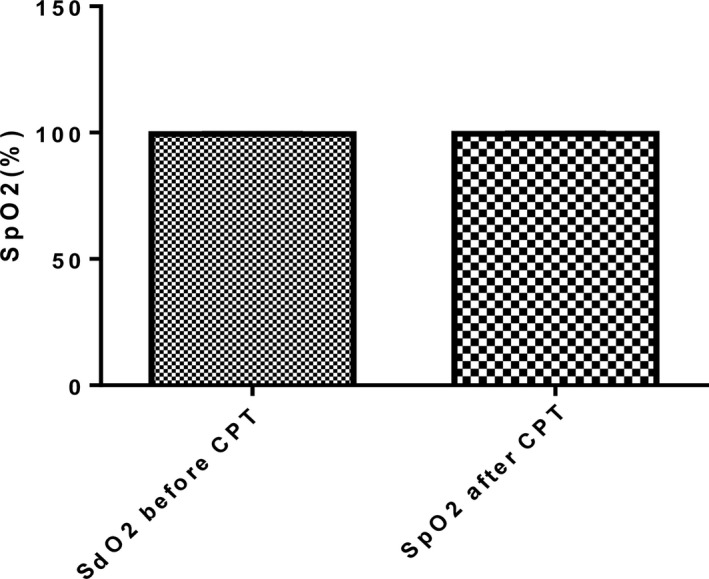
Peripheral capillary oxygen saturation (SpO2) of participants before and after cold pressor test (*n* = 56). SpO2, Peripheral capillary oxygen saturation; CPT, Cold pressor test.

As shown in Figure 3, peripheral capillary oxygen saturation (SpO2) following exposure to CPT was not different (*P* = 0.31) from that at baseline.

### Left ventricular function responses to the cold pressor test

**Figure 4:**
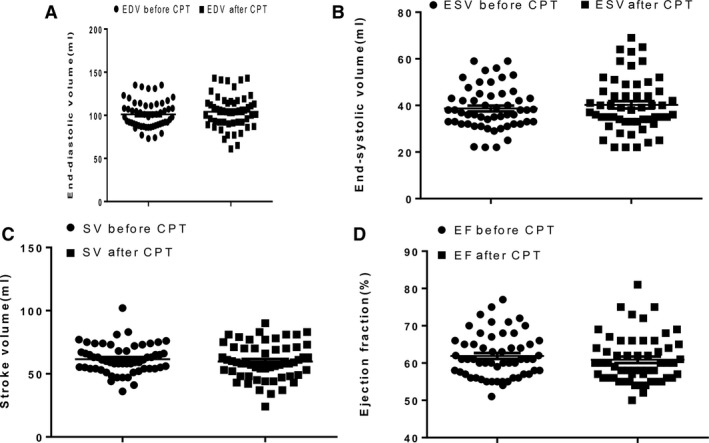
Effect of cold pressor test on left ventricular parameters. EDV, Enddiastolic volume; CPT, Cold pressor test; ESV, Endsystolic volume; SV, Stroke volume; EF, Ejection fraction. n = 56.

**Figure 5:**
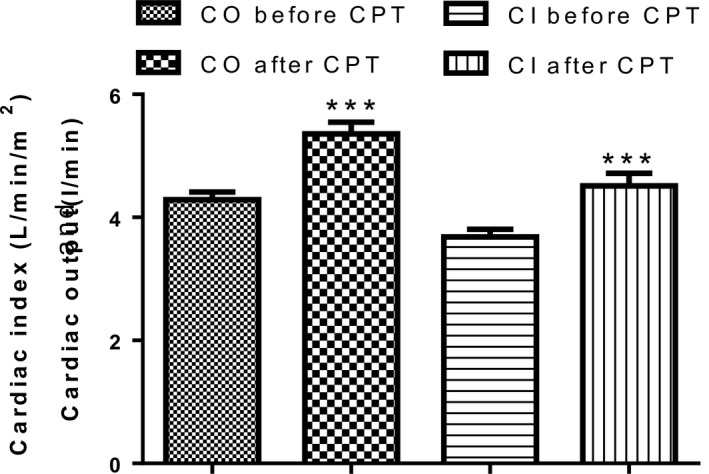
Cardiac output and Cardiac index before and after the cold pressor test (*n* = 56). *** = *P* < 0.0001. CO, Cardiac output; CI, Cardiac index; CPT, cold pressor test.

Following exposure to the CPT, there was no change (*P* = 0.13) in enddiastolic volume (Fig. 4A), endsystolic volume (*P* = 0.26) (Fig. 4B), stroke volume (*P* = 0.29) (Fig. 4C) and Ejection Fraction (*P* = 0.65) (Fig. 4D). The participants’ cardiac output and cardiac index however increased significantly (*P* < 0.0001) from 4.28 ± 0.93 L/min to 5.35 ± 1.44 L/min and 3.68 ± 0.83 L/min per m^2^ to 4.51 ± 1.37 L/min per m^2^ respectively (Fig. 5). Following exposure to the CPT, there was also slight change (*P* = 0.42) in fractional shortening from the baseline value of 33.28 ± 4.5% to 32.55 ± 5.01%.

### Plasma norepinephrine response to the cold pressor test

**Figure 6:**
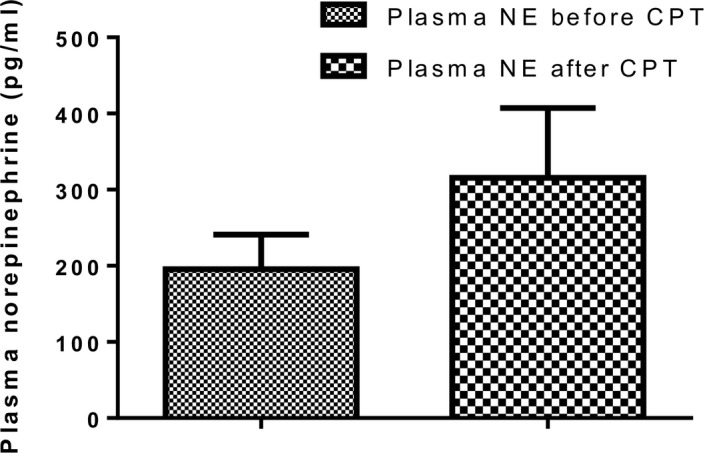
Plasma norepinephrine of participants before and after cold pressor test. NE, Norepinephrine; CPT, cold pressor test.

Plasma norepinephrine (NE) following exposure to CPT was slightly increased (*P* = 0.28) from 195.7 ± 45.4 pg/mL to 315.8 ± 91.45 pg/mL (Fig. 6). Changes in plasma norepinephrine levels (∆NE) were negatively and significantly correlated (*P* < 0.05) with pressor response to the CPT (∆SBP *r* = −0.43;); ∆DBP *r* = −0.40).

## Discussion

Indices of left ventricular systolic function include ejection fraction, fractional shortening and cardiac output. In the present study, the Teichholz method was used to calculate left ventricular volume and ejection fraction. This method calculates left ventricular volume using left ventricular diameter. Its accuracy depends on the accuracy of geometric assumptions about the left ventricular shape. In the Teichholz method, the left ventricle (LV) is assumed a simple ellipsoid with both orthogonal axes being equal.

Stress response is contingent upon intensity, duration and type of stressors. In the present study, the model of sympathetic stress did not produce significant changes in the ejection fraction (EF). This agrees with reports of similar studies by Sagiv et al. 2006 and Wilson and Crandall 2011. The latter researchers found no significant change in ejection fraction in both groups of normotensive older and young adults in their study. In contrast to the present study, Kurtz et al. (2006) reported less than 0.03 unit decrease in EF in normal participants while Bairey et al. (1990) reported a decrease of ≥5% in EF following exposure to the test. It is not clear why the results are conflicting, although it may relate to differences in study population or protocols. The change in EF has been widely used to characterize a normal versus an abnormal response to exercise. The cold pressor test and isometric exercise both activate the sympathetic nervous system, hence they give similar physiological responses (Raoof 2011).

Following exposure to the cold pressor test, enddiastolic volume (EDV) remained unchanged. In contrast, studies using radionuclide ventriculography have reported large increases in enddiastolic volume during increased sympathetic activation (La Gerche and Gewillig 2010). Warburton et al. (2002) suggested that increase in EDV was the dominant means by which cardiac output increased during increased sympathetic activation, especially in welltrained athletes. However, studies using 2D echocardiography (Rowland 2008) and cardiac magnetic resonance (Mayet and Hughes 2003) found little or no increase in EDV.

We report no change in endsystolic volume (ESV) in the present study. The ESV varies in relation to the force of ventricular contraction. Increased sympathetic activation would cause the ventricles to contract strongly and this allows more volume of blood to be ejected, thus the ESV would be lower. However, stress does not have the same effect on ESV in older persons as it has on younger individuals because of increased stiffness of the arteries, decreased cardiac contractility and decline in the response of the heart to sympathetic nervous system (*β*‐adrenergic responsiveness) (Boluyt and Lakatta 2002).

Increase in sympathetic activity leads to an increase in stroke volume (Aliya 2011). However, in the present study, the stroke volume remained unaltered following exposure to cold pressor test. This is similar to the reports of Sagiv et al. (2006) and Wilson and Crandall (2011). This response may be attributed to the vasodilatation mechanism induced by cold pressor test in healthy young adults with normal endothelial function (Monahan et al. 2013). The vasodilatory mechanism keeps the left ventricular volumes and stroke volume during cold pressor test at a low level to nearly the same values obtained at rest (Sagiv et al. 2006). In light to moderate increased sympathetic activation, stroke volume is increased and this results from an increase in venous return leading to the FrankStarling mechanism and increased contractility owing to sympathetic nerve stimulation.

Cardiac output (Mourot et al. 2009) and cardiac index increased following exposure to CPT, just as in the report of Schneider et al. 2003. Our results indicate that the increase in cardiac output was mainly due to changes in heart rate as stroke volume was unaltered in these subjects (Mourot et al. 2009). Our results on cardiac index are in contrast to those of Casiglia et al. 2007, who reported a decrease in cardiac index and cardiac output following exposure to the CPT in waking basal condition (WBC).

Heart rate was seen to increase in all participants following exposure to the CPT, indicative of a sustained autonomic activation. This agrees with reports of previous studies by Gaborit et al. 2012 and Monahan et al. 2013. A massive discharge of catecholamine released from the sympathetic nervous system during cold pressor test activates the *β*‐adrenergic receptors in the sinoatrial node of the heart rate leading to the increase in heart rate (Vaseghi and Shivkumar 2008). However, we recorded only a marginal increase in norepinephrine among the participants in this study.

During the cold pressor test, participants were instructed to avoid performance of Valsalva maneuver and this was assessed by continuous monitoring of their peripheral capillary oxygen saturation (SPO2). Silber et al. (2012) observed that during Valsalva maneuver, there is a reduction in venous return and this results in a transient drop in average blood pressure and pulse pressure. Participants in the current study showed no change in SpO2 following exposure to the cold pressor test, an indication they complied with the instruction. The results observed were therefore strictly due to the effect of the sympathetic challenge and not influenced by the effect of Valsalva maneuver. To our knowledge, our study so far has been the first to monitor SpO2 during exposure to the cold pressor test.

In the present study, the cold pressor test was used as an experimental stimulus to activate the sympathetic nervous system (SNS). Activation of the SNS led to a marginal increase in plasma norepinephrine (pNE) when compared to baseline. Sympathetic stimulation may influence arterial wall mechanics by passively increasing arterial pressure or by changing smooth muscle cell tone (Lydakis et al. 2008). In a study by Izumi et al. (2009) on nonhuman primates, repeated intravenous injections of epinephrine to induce stress cardiomyopathy led to a progressive left ventricular systolic dysfunction with severe hypokinesis in the apical region and hyperkinesis in the basal region on echocardiographic images.

We have demonstrated significant increases in systolic blood pressure (SBP), diastolic blood pressure (DBP) and mean arterial pressure (MAP) following exposure to the cold pressor test. This is consistent with earlier reports (Cui et al. 2002, Elias et al. 2014). Elevation of blood pressure in our results was possibly due to the increase in cardiac output rather than total peripheral resistance (TPR) since the latter remained unaltered; similar to the earlier report by Mourot et al. (2009).

### Limitation of the study

The Simpson's Biplane Method which is a more accurate method to determine left ventricular volumes and ejection fraction could not be used due to the limitation on the time imposed by the duration of exposure to the cold pressor test. The cold pressor test needed to be done in 1 min while the recording of echocardiographic images by Simpson's biplane could only be carried out in a minimum of 3 min.

## Conclusion

Our findings have shown that stress induced by increased sympathetic activation has no effect on the indices of left ventricular function of Ejection Fraction and Fractional Shortening though Cardiac output and the blood pressure parameters were significantly increased.

## Conflict of Interests

There are no conflicting interests.
